# Impacts of Antibiotic Residues in the Environment on Bacterial Resistance and Human Health in Eastern China: An Interdisciplinary Mixed-Methods Study Protocol

**DOI:** 10.3390/ijerph19138145

**Published:** 2022-07-02

**Authors:** Shenghan Cai, Na Wang, Like Xu, Fei Yan, Qingwu Jiang, Xinping Zhao, Wei Wang, Hexing Wang, Lufang Jiang, Wenjuan Cong, Samuel K. Sheppard, Jason Weeks, Barbara Kasprzyk-Hordern, Chaowei Fu, Helen Lambert

**Affiliations:** 1 Population Health Sciences, Bristol Medical School, University of Bristol, Bristol BS8 2PS, UK; shenghan.cai@bristol.ac.uk (S.C.); wenjuan.cong@bristol.ac.uk (W.C.); 2 Key Laboratory of Public Health Safety of Ministry of Education, NHC Key Laboratory of Health Technology Assessment, Shanghai 200032, China; na.wang@fudan.edu.cn (N.W.); fyan@shmu.edu.cn (F.Y.); jiangqw@fudan.edu.cn (Q.J.); xpzhao@shmu.edu.cn (X.Z.); wangwei@shmu.edu.cn (W.W.); wanghexing@fudan.edu.cn (H.W.); jianglufang@fudan.edu.cn (L.J.); 3 Department of Epidemiology, School of Public Health, Fudan University, Shanghai 200032, China; 4 Department of Chemistry, University of Bath, Bath BA2 7AY, UK; lx477@bath.ac.uk (L.X.); bkh20@bath.ac.uk (B.K.-H.); 5 Department of Social Medicine, School of Public Health, Fudan University, Shanghai 200032, China; 6 The Milner Centre for Evolution, Department of Biology and Biochemistry, University of Bath, Bath BA2 7AY, UK; s.k.sheppard@bath.ac.uk; 7 Department of Zoology, University of Oxford, Oxford OX1 2JD, UK; 8 IEH Consulting, Nottingham NG2 6AU, UK; jasonweeks@btinternet.com

**Keywords:** antibiotic resistance, antibiotic exposure, antibiotic-resistant bacteria, cohort study, mixed methods, transmission, wastewater-based epidemiology

## Abstract

Antibiotic resistance is a global health challenge that threatens human and animal lives, especially among low-income and vulnerable populations in less-developed countries. Its multi-factorial nature requires integrated studies on antibiotics and resistant bacteria in humans, animals, and the environment. To achieve a comprehensive understanding of the situation and management of antibiotic use and environmental transmission, this paper describes a study protocol to document human exposure to antibiotics from major direct and indirect sources, and its potential health outcomes. Our mixed-methods approach addresses both microbiological and pathogen genomics, and epidemiological, geospatial, anthropological, and sociological aspects. Implemented in two rural residential areas in two provinces in Eastern China, linked sub-studies assess antibiotic exposure in population cohorts through household surveys, medicine diaries, and biological sampling; identify the types and frequencies of antibiotic resistance genes in humans and food-stock animals; quantify the presence of antibiotic residues and antibiotic resistance genes in the aquatic environment, including wastewater; investigate the drivers and behaviours associated with human and livestock antibiotic use; and analyse the national and local policy context, to propose strategies and systematic measurements for optimising and monitoring antibiotic use. As a multidisciplinary collaboration between institutions in the UK and China, this study will provide an in-depth understanding of the influencing factors and allow comprehensive awareness of the complexity of AMR and antibiotic use in rural Eastern China.

## 1. Background

Since the mass production and clinical application of effective antimicrobials in the 1940s, their effectiveness in treating bacterial infection has been challenged by a gradual increase in antimicrobial resistance (AMR) worldwide [1,2]. The ever-increasing amount of antibiotics consumed and disseminated provides constant selection pressure for the development of resistant bacterial strains. Growing evidence shows that commercial pressures and persistent misuse of antimicrobial agents in humans and animals have fuelled the spread of antimicrobial resistance globally [3,4,5]; bacterial resistance to available antibiotics threatens human and animal lives, especially among low-income and vulnerable populations in less-developed countries [6,7]. Existing literature has identified a range of influencing vectors and possible intervention strategies relating to bacterial resistance in humans [8,9], including the effects of antimicrobial consumption, population exposure to antibiotic resistance genes (ARGs), and antibiotic residues via livestock, food, and the environment. Initiatives such as institutional regulation and the surveillance of resistant pathogens and antimicrobial use, as well as public campaigns about the safe and effective use of antibiotics, are being promoted to reduce the burden of antimicrobial resistance globally [4,5,8]. Local knowledge and approaches are crucial to unravel the problem of antibiotic resistance, as patterns of resistance vary locally and the development of effective interventions requires tailoring to specific socioeconomic, cultural, and health system settings.

There is a high burden of AMR in China [10,11], with substantial evidence showing that antibiotic misuse, from clinical medical practitioners’ overprescribing to self-treatment or preventative medication in daily life and agricultural production activities, have fuelled resistance [12,13,14]. To date, the majority of existing studies have focused on depicting the behavioural, social-structural, and biochemical dimensions of antibiotic resistance. Studies have shown that health practitioners tend to overprescribe antibiotics as a result of economic considerations, reputational pressure, and patient demands [15], while the public is less educated on the appropriate use of antibiotics [16,17,18]. Self-medication, such as over-the-counter purchasing and reuse of leftover drugs, is also an important contributor to unnecessary antibiotic use. In addition to clinical factors, socioeconomic and cultural factors also play key roles in shaping individual knowledge and behaviour on the consumption and dissemination of antibiotics [19].

Population exposure to antibiotics is not limited to clinical use, as antibiotic residues can be transmitted through the living environment. While the biological drivers of antibiotic resistance are well explored, human–microbial interactions are less well understood. Although microbial ecosystems among human, animal, and environmental components differ, the environment acts as a bridge in the accumulation and transmission of antibiotic-resistant bacteria (ARBs) and ARGs between humans and animals, particularly through food sources and excrement. The widespread use of antibiotics does not only put pressure on both humans and animals, but also affects the environment, which, in turn, may result in further selection and circulation of ABRs among different microbial ecosystems. Researchers have investigated resistance genes in humans, animals, and the aquatic environment, but only a few studies have explored transmission across different environmental compartments [6].

Policy interventions and surveillance of antibiotic use and ABRs mainly focus on pharmaceutical prescribing within the healthcare system and in retail outlets. National policies and regulations, together with the national surveillance system, provide generic guidance on antibiotic use; meanwhile, in practice, multiple departments at provincial and lower levels may design or specify detailed and context-related policies or guidelines in accordance with the local situation across healthcare, food and drug administration, the environment, and agriculture. Thus, although several studies have demonstrated reductions in the overall use of antibiotics following national interventions on antibiotic use and healthcare reforms, their effects on local patterns of antibiotic prescription and use have been little-documented, particularly in rural settings, outside the public healthcare system, and in relation to food safety and industry.

To achieve a comprehensive understanding of the situation and management of antibiotic use and environmental transmission in Eastern China, this protocol describes a study to document human exposure to antibiotics from major direct and indirect sources, as well as its potential health outcomes. Our mixed-methods approach addresses both microbiological, pathogen genomics, epidemiological, geospatial, anthropological, and sociological aspects. Implemented in two rural residential areas in two provinces in eastern China, linked sub-studies will assess antibiotic exposure in population cohorts through household surveys, medicine diaries and biological sampling; identify the types and frequencies of ABR genes in humans and food-stock animals; quantify the presence of antibiotic residues and ABR genes in the aquatic environment, including wastewater; investigate the drivers and behaviours associated with human and livestock antibiotic use; and analyse the national and local policy context, to propose strategies and systematic measurements for optimising and monitoring antibiotic use.

## 2. Objectives and Study Design

### 2.1. Aim and Objectives

This study aims to investigate direct and indirect human exposure to antibiotics and the subsequent potential health effects; the transmission of ABR in the aquatic environment, animals and humans; and current policy provision and implementation relating to ABR through a set of linked sub-studies, in order to provide the evidence needed to facilitate effective antibiotic stewardship and identify ways to monitor and control potential health hazards.

The study objectives are:

1. To comprehensively assess the exposure levels of humans to antibiotics from clinical and environmental sources.
2. To investigate the types and abundance of ARGs; assess the effects of antibiotic exposure on resistance genes; and explore possible pathways of transmission of ABR genes among livestock, the environment, and the population.
3. To describe the spectrum of antibiotics and usage patterns in representative Chinese populations, and explore the relationship between the use of antibiotics and exposure levels of humans to antibiotic residues and antibiotic-resistant genes.
4. To assess comprehensive spatiotemporal community-wide public exposure to antibiotics (public intake vs. total environmental burden) and the resulting ABR using wastewater-based epidemiology (WBE).
5. To understand the current policy context relating to antibiotic usage and systems for monitoring ABR; to explore the implementation of policies to limit antibiotic use in the health, agricultural, and environmental sectors; and to identify strategies to improve monitoring and optimise antibiotic use.

### 2.2. Study Setting

Due to its dense population, intensive livestock breeding industry, and high antibiotic use, East China is a well-recognised hotspot of antibiotic consumption and ABR. This study focuses on two economically and epidemiologically distinct rural residential areas in Eastern China—Village A of Zhejiang Province and Village B of Jiangsu Province. Both sides are economically comparable, but the former is a relatively remote setting, whereas the latter is located near a large city with convenient access to its healthcare system. Differences between settings, antibiotic use, local regulations, water environment, and poultry and aquaculture practices in the two communities provide opportunities for comparison. The study sites were selected to facilitate the use of existing epidemiology study cohorts to explore the potential long-term effects of antibiotic use and exposure to environmental antibiotic residues and resistance genes.

Study site A has a total population of 2071. The local topography is high in the west and low in the east; the source of surface runoff in the dry season is via a lake system, and during the rainy season, it is mainly via a mountain water system. Water in the local community is supplied by a centralised water plant. Residents’ drinking water is mostly via tap water, but wells and ponds are used for washing and laundry. The treatment of rural domestic water discharge is “one pipe connected to the end”, as bathroom sewage, kitchen sewage, washing sewage, and bathing sewage are all collected by the same pipe system and delivered to the sewage-treatment terminal. Aquaculture farming is the main local business, and the total scale of ponds is around 1.33 million square metres. Specialised ecological channels surrounding the farm ponds are used to transfer fishery farming discharge/wastewater to the specialised treatment facility. Although residents cultivate small-scale crops and poultry for self-consumption, their main source of food for daily consumption is the grocery store and supermarket.

Study site B is a community with around 1978 residents in an area that traditionally has a high incidence of liver cancers. Local housing follows a linear pattern alongside irrigation canals, and the housing and canals are interlaced within the residential area. Water in the irrigation canals is stagnant, filled with precipitation and residential domestic water discharge as the two main water sources; excrement from poultry from local households is directly discharged into the open irrigation canals. The local population has high exposure to irrigation canal water, as it is used by the surrounding residents for their small-scale poultry and aquatic farming, the products of which are consumed by the residents directly; it is also used during food preparation. Even though tap water and well water are the main sources of drinking and cooking water, the groundwater level is less than three metres deep.

Wastewater Treatment Plants (WWTPs) located adjacent to study sites A and B will be included to explore public exposure to antibiotics in larger communities using the wastewater-based epidemiology (WBE) approach. Due to the complexity of local economic structure, sources of antibiotics in WWs include hospitals, residential areas, animal farming and industrial districts. A total of 6 WWTPs representing different scales will be selected, which serve population sizes ranging from 12,000 to 634,000 in the local area.

### 2.3. Participants

To measure local population exposure to antibiotics, all adults living in the study areas for no less than three months and able to give consent to participate in the study will be eligible for recruitment. All participants will be invited to complete the survey and record daily medicine intake in the household for a one-year period.

All village clinics, township health centres/hospitals, and county hospitals that serve the two areas will be included in the formal research. At each study site, physicians and health professionals from different departments of county-level and community-level health institutes, especially professionals overseeing the management of clinical antibiotic use, will be invited for interview. In-depth interviews will be conducted with relevant experts, including officers in hospital or pharmacy management departments, managers from the local health committees, medical product administration, and managers from county-level hospitals and township health centres. Technicians and related personnel in county hospital labs will also be included in the formal research. Local farmers, workers from retail pharmacies and veterinary pharmacies and veterinary technicians will also be interviewed.

### 2.4. Study Design

#### 2.4.1. Transmission of ABR Genes, Environmental Antibiotic Residues, and ABR Bacteria

Microbiology and genomics analysis will include collection of human (urine and faecal), animal (meat/gut, faecal, fodder), and environmental (water and wastewater) samples from local residential and occupational populations, livestock, and aquatic products. ARG detection will be used to explore the distribution of ABR genes in the environment and animals, as well as the potential transmission from environmental and animal sources to the residents.

Environmental assessment will focus on sampling different types of water, which includes household drinking water such as tap water, well water, and bottled water, and wastewater from treatment plants, farms, irrigation canals, and the water surface surrounding the residential areas. The objective is to assess potential environmental exposure to antibiotic residues and ABR genes in the water and its association with local population exposures—which will be measured via urine samples from households in the two study areas.

#### 2.4.2. Antibiotic Use in Healthcare Facilities and Communities

To describe the spectrum and patterns of antibiotic use in the population and livestock, and explore the relationship between antibiotic use, ABRs, and human health in the daily living context, village doctors, patients, and residents from the two study sites will be recruited for semi-structured interviews. Information on residents’ antibiotic use will be collected both inside and outside the healthcare system. Clinical prescription data will be obtained through electronic patient records from hospitals, but the records do not contain comprehensive information on antibiotic use at lower-level health facilities or where antibiotics are purchased over the counter from retail pharmacies.

A survey questionnaire and a medicine diary (pilot tested—see Supplement) will therefore be used to collect and record additional relevant information. Local community health professionals will work with researchers to collect survey data. A structured self-administered diary will be used to collect medicine use (antibiotics and chronic disease medicine) and individual health status in each participating household over a 12-month period. In addition, we will conduct focus-group interviews with residents to collect information on risks of exposure to antibiotics through food, water and medicine, awareness of antibiotics and AMR, and choice of healthcare services.

Wastewater-based epidemiology will be used to evaluate antibiotic usage at a community level. Wastewater contains a complex mixture of chemical substances including human excretion products of external or internal bodily origin resulting from exposure to xenobiotics, such as antibiotics and infectious agents, and internal processes, such as specific disease or exposure-linked proteins, genes, and metabolites. The quantitative measurement of these specific endo and exogenous residues pooled by the sewerage system serving different communities can provide valuable evidence of the quantity and type of xenobiotic chemical agents to which the surveyed population was exposed and can inform on the effects of this exposure. Quantification of antibiotics and their metabolic residues can provide information of daily ‘true’ antibiotic usage by whole communities served by individual wastewater treatment works [20,21].

**Figure 1:**
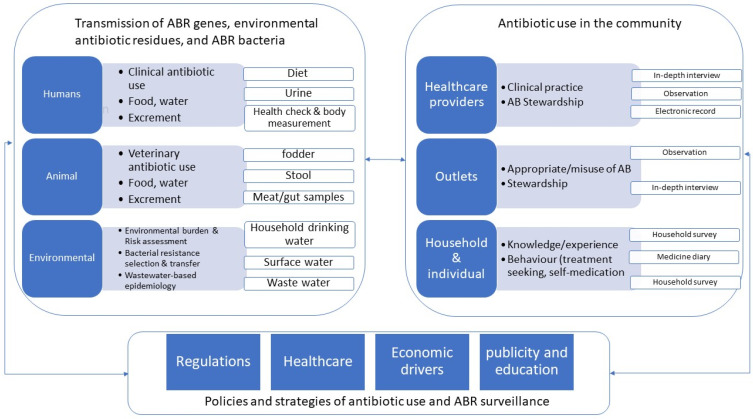
Description of study design and quantitative and qualitative data collection.

The overall study design was illustrated in Figure 1.

#### 2.4.3. Policies and Strategies of Antibiotic Use and ABR Surveillance

Healthcare facilities such as village clinic, township health centre and county/provincial hospital, as well as retail pharmacies provide antibiotics to community members. Clinical use and self-medication are the two important dimensions of antibiotic consumption. National, provincial, county/township level regulatory procedures and policies will be collected and analysed to depict the structural setting and institutional regulation of antibiotic use and ABR surveillance. We will recruit patients, medical practitioners and relevant managers for interviews from hospitals and private clinics. Patient interviews will focus on outpatients in hospital Respiratory, Gastroenterology, Surgical, Paediatrics departments with high antibiotic prescribing rates. Retail pharmacy is closely linked to self-medication on both human and animal; researchers will visit local pharmacies to observe and interview pharmacy staff. Customers in retail pharmacies and residents who participated in the survey will also be recruited for interview.

Drivers and practices regarding antibiotic use and ABRs in communities and livestock will be explored with health professionals and key informants through in-depth individual and focus group interviews, to understand relationships between existing policies and patterns of behaviour on antibiotic use. In addition, local farmers, fodder retailers, and township agricultural technology stations will be also included in formal interviews.

## 3. Methods

### 3.1. Biological and Environmental Sampling

Collection of urine samples, anthropometric measurements (body height, body weight, and waist circumference) and biochemical tests (urine creatinine) will be performed at the time of the annual health check and household survey. Spot urine samples will be collected from each participant during the investigation and stored at −20 °C at the local health service centre. All samples will be transported to the lab within one month after collection and then stored at −80 °C for future analysis of antibiotics. Two-hundred and twenty-five (225) flesh samples of fish will be collected from local fish farms and households. A total of 115 food samples from meat, market produce, and abattoirs will be collected and analysed for antibiotics.

In all study areas, we will conduct geospatial mapping of observed surface water using a GPS-enabled drone. Two detailed maps of the selected study sites will be drawn and measured to depict the geographic distribution of municipal sewage discharge, main rivers, and cultivation industry (including aquaculture farms and chicken farms). The mapping area will also include local resident households, from which 20 households per site will be selected randomly. Drinking water samples, cooking/well water samples, and surface water samples from the nearest location will be collected from each household. For water samples collected from the river, the sampling location will be at both ends of the river, and also at each site of given distance along the river. The collection of water samples will be repeated during different seasons (dry and wet) with two parallel samples collected at each site. GPS coordinates will ensure the repeatability of geographic location of sites. By connecting the wastewater sample with medicine diary, this will enable us to explore the connections between residents’ antibiotic use and ABR residues in the wastewater sample.

Wastewater sampling will be conducted during different seasons, along with the above-mentioned environmental sampling in the villages. Influent samples will be collected over 7 consecutive days for each campaign. In order to obtain a representative influent sample, a flow-proportional composite sampling strategy will be adopted by using an auto-sampler to collect influents at regular time intervals for 24 h. The method used for the quantification of antibiotics in wastewater will follow a previous study [22]. A total of 56 antibiotics and 26 major metabolites will be targeted for wastewater sample analysis. The analytes selected cover all major antibiotic groups used for both human and veterinary medicines, including aminoglycosides, azoles, β-lactams, macrolides and lincomycin, nitrofurans, phenicols, quinolones, sulfonamides, and trimethoprims, and tetracyclines.

### 3.2. ABR Genes and Transmission

The genomic DNA of the samples from wastewater will be extracted and sequenced [23,24]. Extraction will be conducted by using the sample preparation protocol developed by Chopyk, J. It will be linked to residents and households to identify the possible drivers at the local level. The homology of ABR genes from animal samples and the professionally exposed population (aquaculture personnel, salespersons, and stockbreeding and slaughterhouse workers) will be analysed and possible pathways of ABR gene transmission determined. The metagenomics of human gut microbiota, aquatic environment and livestock microbiota will be carried out if necessary. Selected typical multiple-drug-resistant (MDR) bacteria, e.g., MRSA, will be isolated and their genomes will be analysed to understand the transmission of MRSA among livestock and professionally exposed populations.

In addition to metagenomic sequencing, representative ABR genes will be selected for quantitative PCR (qPCR) analysis. The selection of ABR genes should correspond to the antibiotic groups analysed. The ABR gene spectrum of microbiome in the population, animals, and the environment will be analysed and integrated with the characteristic of antibiotic exposure.

#### 3.2.1. Chemical Analysis of Antibiotics in the Environment and Urine

Solid-phase extraction (SPE) will be conducted using a sample preparation protocol developed by Holton et al. [22]. Briefly, water samples will be filtered through a GF/F glass fibre filter (0.7 μm, Whatman, Maidstone, UK) and the filtrates were loaded under vacuum onto pre-conditioned Oasis HLB cartridges (60 mg, Waters, Manchester, UK). The cartridges will be then conditioned and equilibrated. After loading, cartridges will be dried under vacuum, sealed with parafilm and stored at −20 °C freezer, until being eluted and tested. An analytical method previously established for 21 antibiotics using two-dimensional ultra-performance liquid chromatography coupled with quadrupole time-of-flight mass spectrometry (UPLC-Q/TOF MS) [25] will be expanded to 38 antibiotics and 3 of their metabolites by optimizing the conditions of the sample pre-treatment, chromatographic separation, and mass spectrometer. UPLC-Q/TOF MS will be used to detect their presence in samples and combined with questionnaire data on recent antibiotic use to assess total exposure to antibiotics from multiple sources. For the analysis of antibiotics presented in tap, well, river, and aquaculture water, results will be presented as concentration levels (ng/L or μg/L) to directly compare the spatiotemporal variations across the sampling sites and seasons. For food (e.g., meat, egg, aquatic feed, etc.) samples, results will be presented as concentration levels (ng/g or μg/g) to assess the antibiotic residue levels.

#### 3.2.2. Chemical Analysis of Antibiotics in WWTPs and WBE Back-Calculations

Raw data originally generated from the instrument will be integrated using MassLynx software packages (Version 4.0, Waters, Manchester, UK), and results will be presented as concentration levels (ng/L or μg/L) in wastewater influents. Daily mass loads of antibiotics will be calculated by multiplying the concentrations of the analytes by the daily flow rate (L/day) and then normalised by the population size of the served communities (number of people contributing to wastewater analysed). Human metabolism and sewer loss will be incorporated into the calculation to correct the antibiotic consumption levels in the community. Final data will be presented as (mg/day/1000 people) and will further be compared with the medicine diary data to fully understand the spatiotemporal public exposure vs. the environmental burden of antibiotics.

#### 3.2.3. Quality Assurance in Chemical and Biological Analysis

After the sample collections are completed, samples will be stored on ice until transferred to the laboratory. Deuterated (stable isotope-labelled) standards will be spiked on-site and samples will subject to solid-phase extraction. The analytical method for antibiotic quantification will be performed using a Waters ACQUITY UPLC^TM^ system coupled with a Xevo TQD-ESI Mass Spectrometer. Mobile phase and matrix quality controls will be used to monitor analyte calibration and physical–chemical behaviour between the varying sample compositions. Physical sample duplicates will be prepared and analysed to improve overall confidence.

#### 3.2.4. Structured Household Survey

Household registration records from two sites will be used as the overall sampling frame, in which all long-term residents (living in the area for at least three months at the time of survey) will be recruited. Residents who were not documented in registration records but meet this criterion will also be recruited in the study. Survey will be carried out during the local annual health examination—a free health service funded by NRCMS medical insurance scheme and provided by the local community to all residents. If residents were absent from the health check-up, they will be contacted afterwards by the researcher, assisted by local practitioners and community workers.

A trained survey team will administer the questionnaire to those residents who consent to being recruited following their physical examination and collection of urine samples. Representatives of the research team will be present to ensure quality control. The structured questionnaire will collect detailed information from respondents, including demographic information, medical history, personal lifestyle, physical activity, dietary preference, self-evaluated health status (using EQ5D-5L questions), and attitude and knowledge on antibiotic use (adapted version based on WHO ABR awareness survey). Those residents who do not attend the annual health examinations will be contacted subsequently by the village doctor on their regular household visits. Doctors and researchers will seek non-attendees’ consent to be recruited to complete the survey and provide relevant samples.

Statistical tests will be performed in SAS or SPSS software. The dietary exposure to antibiotics will be estimated by combing data on antibiotic contamination in foods and drinking water, along with the information on dietary consumption. Multiple statistical models, including the logistic regression model and linear regression model, will be used to explore the potential effects of exposure to antibiotics from multiple sources on obesity, diabetes mellitus, asthma, and IBD in the cohort, by examining the associations between their occurrence and antibiotics in urine, after adjustment for potential confounders.

#### 3.2.5. Medicine Diary

During initial recruitment, all participants will be asked to provide consent to being followed up for the data collection of their medicine diaries. Data collection will continue for 12 months to capture possible seasonal variations. A structured and self-reported form has been developed to collect information on demographic backgrounds, chronic disease, and daily use of medicine of all members in the household.

Those who consent will receive training on how to complete the medicine diary from local village doctors. Data on medicine use for each household member are recorded weekly by participants using the structured form to capture the types, reasons, sources (prescribed, self-purchased, or leftover/home-storage), frequencies, doses, side effects, and other details of both antibiotic use and use of medicines for long-term conditions. Considering the length of the data collection period, accessibility of the sites, and dialects, local village doctors will be trained to provide consultation to the participants and assist with data collection. Medicine diaries will be collected by the village doctor from households, while researchers will collect each batch of medicine diaries, carry out onsite quality control, and analyse the data monthly. Village doctors will also contact participants regularly through telephone calls as a reminder of the monthly diary collection. During the period of data collection, all pharmaceutical packages of the medicine consumed by the participants will be collected side-by-side. Modest remuneration in kind will be provided at the time of diary collection to acknowledge participants’ time and effort.

All data will be double-entered into EpiData software (version 3.1, “The EpiData Association” Odense, Denmark). Qualitative data will be coded and analysed using the inductive method. The results will be correlated to the consumption mode of antibiotics based on the medicine diary. To ensure data quality, all data will be recoded and double-entered by a special team of graduates. The investigators will provide a detailed interview manual and undertake onsite quality control.

#### 3.2.6. Electronic Medicine Record

One year of medicine records, including pilot and pandemic period, in local healthcare facilities (village clinics and township health centres) will be collected. Completed medicine diaries will then be used to compare with individual patient records from the local village clinic every three months in the following year. The research team will review all entries in the electronic records, and the records will be matched with participants using their medical insurance number and ID number. This data will be anonymised and utilised to compare with self-reported medicine diaries and calculate the rates of antibiotic prescription and self-medication.

#### 3.2.7. In-Depth Interviews

Qualitative data will be collected using standardized and semi-structured topic guides across study sites. Interviews will be audio recorded with pre-consent. Data collection will stop once data saturation has been reached. Participants will include farmers, patients, doctors, administrators, and managing staff (detailed list, number, and inclusion criteria of stakeholders; see Supplementary Materials).

Interviews with convenience-sampled outpatients (20 per study area) and doctors (20 per study area) will take place in the recruitment village clinic, township health centre, private clinics, andprivate hospitals. Two topic guides will be used to investigate their experience of consultation and diagnosis; knowledge and attitudes on antibiotics; and understanding of resistance and regulation. In addition, a series of COVID-related questions will be used to investigate the influence of the pandemic on the prescription and use of antibiotics.

The administrator and managing staff from local government will be recruited to explore the detailed regulation and surveillance of antibiotic use, residue, and resistance. All administrators and pharmacists whose main responsibility relates to stewardship in local regulation departments and recruited hospitals will be interviewed to understand the accessing of antibiotics, possible economic drivers, and institutional regulation of antibiotic use. Based on a pilot study and the local staffing structure, 10–15 interviews will be conducted per study area. Pharmacy and veterinary pharmacy workers located within the areas of study will also be recruited. Relevant managing staff (5 per study area) and poultry and aquatic workers (10 to 15 per study area) will be interviewed to collect their knowledge and practice of antibiotics in farming activities.

#### 3.2.8. Community Focus-Group

In each study site, based on the population age and gender structure, 2 to 3 focus groups of 20 to 30 residents who have completed the household survey and medicine diary, with different socioeconomic statuses, will be recruited. The topic guide will cover recent experience of illness, experience of healthcare services, accessing drugs, attitudes and knowledge on antibiotic use, perceptions of resistance, and proper use.

For qualitative data, coding will be undertaken independently by at least two researchers for each sub-group of informants in NVivo v.12 and a common coding framework developed. Following the identification of emergent themes, further coding across groups will be undertaken to generate generalizable findings. Interview data will be used to characterize antibiotic use and systems for ABR monitoring in our study regions, including the allocation of incentive, human resources, technologies, and information, and the building of supervision systems. The implementation processes and outcomes of these policies and regulations, including how health and agricultural sectors aim to reduce the use of antibiotics, supervision tools, incentives for changing behaviour, and outcomes, will be assessed. Recommendations and strategies for optimizing antibiotic use and ABR monitoring systems in lower-level hospitals and rural health facilities will be informed by the key findings and international comparisons.

Data gathered during focus group discussions will be analysed using systematic thematic coding in NVivo to explore experiences of treatment-seeking and antibiotic use, including self-medication; drivers and practices regarding antibiotic use; and ABR in communities and livestock.

#### 3.2.9. Synthesis

Considering the documented types, frequencies, and doses of antibiotics consumed, total antibiotic consumption will be calculated and compared between different subgroups by using nonparametric tests methods. The qualitative and quantitative social science and microbiological datasets will be linked in order to perform more complex analyses. Consumption levels of antibiotics from medicine diaries and household survey data, as well as from electronic patient records, where available, will be correlated with environmental sample analyses to triangulate evidence on antibiotic exposure (see above). The analysed data will be used to develop a hierarchical risk-based framework for structured decision-making and to identify key hazard sites and pathways for the development of future interventions.

## 4. Implementation

### 4.1. Biological and Environmental Sampling

A total of 1520 urine samples were collected in July to August 2020 at the time of the household survey. One-hundred and sixty-two fresh samples of food, including vegetables, meat, fish, and eggs were collected from local households.

With a GPS-enabled drone, detailed maps of the two selected study sites were drawn and 10 households were selected randomly in each site. A total of 822 water samples, including drinking water, well water, pond water, and surface water, were collected. The collection of water samples was repeated during different seasons (dry, i.e., January 2021, and wet, i.e., July to August 2020) with two parallel samples collected at each site. Moreover, 84 wastewater samples were collected during different seasons, along with the above-mentioned environmental sampling in the villages.

### 4.2. ABR Genes and Transmission

The genomic DNA of the water samples has been extracted and sequenced, but the analysis of ABR genes is still ongoing. Selected typical MDR bacteria, e.g., MRSA, have been isolated, and their genomes are also under analysis.

### 4.3. Chemical Analysis of Antibiotics in the Environment and Urine

Using UPLC-Q/TOF MS, 25 antibiotics and two of their metabolites have been detected in the above-mentioned water samples. However, the detection of antibiotics in food samples has not been completed yet.

### 4.4. Structured Household Survey

Household registration records from two sites were used as the overall sampling frame. A field investigation was carried out from July to August 2020, and 1521 subjects were recruited into this study.

### 4.5. Medicine Diary and Electronic Medicine Record

From July 2020 to June 2021, 783 subjects provided the medicine diary, monthly, from two sites. All data were double entered into the EpiData software (version 3.1, “The EpiData Association” Odense, Denmark). One year of medicine records in local healthcare facilities (village clinics and township health centres) were collected and are being matched with participants using their medical insurance number and ID number.

### 4.6. In-Depth and Community-Focus-Group Interviews

Qualitative data were collected using standardised and semi-structured topic guides. In total, 103 individuals were interviewed deeply, and 13 focus groups of 67 subjects have been conducted since 2019. For qualitative data, coding was undertaken independently by at least two researchers for each sub-group of informants in NVivo v.12 with a common coding framework.

## 5. Discussion

Using two villages in Eastern China as an example, this proposal plans to study the diverse and complex problem of antibiotic use and resistance. It proposes the utilisation of a ‘One health’ approach to provide better evidence and a broader understanding of the environmental, community, economic, and healthcare drivers and burdens of ABR based on a systems perspective that recognises that interactions between these areas is urgently needed, as are evaluation tools to measure the effectiveness of different ABR-reducing intervention strategies. Due to a dense population, an intensive livestock-breeding industry, and massive antibiotic use, Eastern China is a key region for controlling antibiotic use and ABR. Our research aims to bridge these key evidence gaps and strengthen disciplinary and methodological research skills, through a set of closely linked projects that will generate the holistic knowledge which is needed to design, deliver, and monitor targeted strategies to limit ABR in China and comparable settings.

New knowledge and robust evidence will be gained regarding the transmission pathways of antibiotic resistance between aquatic environments, animal food sources, and workers who are professionally exposed to these sources. The monitoring approach that will be developed through sampling in the aquatic environment will provide a potential tool for use in the research and monitoring of ABR transmission elsewhere. The utilisation of sophisticated assays to detect antibiotic residues in population cohorts with well-documented antibiotic use will produce rich data on the relationship between direct and indirect antibiotic exposure and the associated long-term health risks. Analysis of wastewater from these populations will contribute to appraising the potential of this novel approach in monitoring community public health. Our research findings on antibiotic use from this project will also produce a uniquely detailed, representative data set on when, why, and where antibiotics are prescribed and purchased in low- and middle-income countries’ community settings. This will be valuable for health systems researchers and social scientists (including anthropologists, sociologists, psychologists, and economists) working in global health, while our work on policy implementation will provide insights into the anticipated and unplanned consequences of policy and regulatory changes that aim to limit the prescription and consumption of antibiotics and other medicines.

### Patient and Public Involvement

Local stakeholders are invited for meetings to discuss the preliminary study results. There is a possibility of community meetings to share/provide feedback on the study results with local residents and stakeholders. The findings from the study will be disseminated in peer-reviewed journals and will also be presented at scientific conferences.

## 6. Conclusions

This multidisciplinary collaboration between institutions in the UK and China will provide an in-depth understanding of the influencing factors and allow comprehensive awareness of the complexity of AMR and antibiotic use in rural Eastern China.

## Data Availability

This study will generate multiple datasets. Following study completion, datasets will be uploaded and stored in the Research Data Storage Facility (RDSF), the University of Bristol institutional repository, or other appropriate open-access repositories (e.g., the UK Data Service https://ukdataservice.ac.uk/ (accessed on 22 February 2022) for clinical data, or the NERC Environmental Data Service https://eds.ukri.org/ (accessed on 22 February 2022) for environmental data). Outputs from the study, including the presentation of supporting data, will be published on an open-access basis and will include weblinks to data location in the relevant data repository. RDSF permits configuration for both restricted and open access. Most datasets will be made available on an open-access basis. Datasets containing sensitive data (e.g., medicine diaries, qualitative data containing potentially identifiable geographical or personal information) will not be made public, but will be made available from the corresponding author on reasonable request.

## Supplementary Materials

The following supporting information can be downloaded at: https://www.mdpi.com/article/10.3390/ijerph19138145/s1, Table S1: Medicine Diary and family member antibiotic use records; Table S2: List of potential interviewees and proposed data collections.

## Abbreviations

AB	Antibiotic
ABR	Antibiotic resistance
AMR	Antimicrobial resistance
ARGs	Antibiotic resistance genes
DNA	Deoxyribonucleic acid
GIS	Geographical Information System
GPS	Global Positioning System
ICC	Intra-class coefficient
MDR	Multiple-drug-resistant
MRSA	Methicillin-resistant *Staphylococcus aureus*
qPCR	Quantitative polymerase chain reaction
UPLC-Q/TOF MS	Ultra-performance liquid chromatography coupled with quadrupole time-of-flight mass spectrometry
WBE	Wastewater-based epidemiology
WHO	The World Health Organization
WW	Wastewater
WWTPs	Wastewater Treatment Plants (WBE)

## References

[1] Davies J., Dorothy D. (2010). Origins and evolution of antibiotic resistance. Microbiol. Mol. Biol. Rev. MMBR.

[2] Roca I., Akova M., Baquero F., Carlet J., Cavaleri M., Coenen S., Cohen J., Findlay D., Gyssens I., Heure O.E. (2015). The global threat of antimicrobial resistance: Science for intervention. New Microbes New Infect..

[3] Ventola C.L. (2015). The antibiotic resistance crisis: Part 1: Cause and threats. P T A Peer-Rev. J. Formul. Manag..

[4] WHO Global Action Plan on Antimicrobial Resistance 2016. https://www.who.int/publications/i/item/9789241509763.

[5] WHO (2022). Antimicrobial Resistance (Antimicrobial Resistance) [World Health Organization Fact Sheets]. https://www.who.int/news-room/fact-sheets/detail/antimicrobial-resistance.

[6] Cars O., Högberg L.D., Murray M., Nordberg O., Sivaraman S., Lundborg C.S., So A.D., Tomson G. (2008). Meeting the challenge of antibiotic resistance. BMJ.

[7] Alsan M., Schoemaker L., Eggleston K., Kammili N., Kolli P., Bhattacharya J. (2015). Out-of-pocket health expenditures and antimicrobial resistance in low-income and middle-income countries: An economic analysis. Lancet Infect. Dis..

[8] Laxminarayan R., Malani A. (2007). Extending the Cure: Policy Responses to the Growing Threat of Antibiotic Resistance.

[9] Levy S.B. (2002). Factors impacting on the problem of antibiotic resistance. J. Antimicrob. Chemother..

[10] Heddini A., Cars O., Qiang S., Tomson G. (2009). Antibiotic resistance in China—a major future challenge. Lancet.

[11] Liu J., Fang Z., Yu Y., Ding Y., Liu Z., Zhang C., He H., Geng H., Chen W., Zhao G. (2021). Pathogens distribution and antimicrobial resistance in bloodstream infections in twenty-five neonatal intensive care units in China, 2017–2019. Antimicrob. Resist. Infect. Control.

[12] Wang J., Wang P., Wang X., Zheng Y., Xiao Y. (2014). Use and prescription of antibiotics in primary health care settings in China. JAMA Intern. Med..

[13] Wang C., Huttner B.D., Magrini N., Cheng Y., Tong J., Li S., Wan C., Zhu Q., Zhao S., Zhuo Z. (2020). Pediatric antibiotic prescribing in China according to the 2019 World Health Organization Access, Watch, and Reserve (AWaRe) antibiotic categories. J. Pediatr..

[14] Wang N.C., Liu Y. (2021). Going shopping or consulting in medical visits: Caregivers’ roles in pediatric antibiotic prescribing in China. Soc. Sci. Med..

[15] Chen M., Kadetz P., Cabral C., Lambert H. (2020). Prescribing antibiotics in rural China: The influence of capital on clinical realities. Front. Sociol..

[16] Wang X., Zhou X., Hesketh T. (2016). Massive misuse of antibiotics by university students in China: A cross-sectional survey. Lancet.

[17] Chang Y., Chusri S., Sangthong R., McNeil E., Hu J., Du W., Li D., Fan X., Zhou H., Chongsuvivatwong V. (2019). Clinical pattern of antibiotic overuse and misuse in primary healthcare hospitals in the southwest of China. PLoS ONE.

[18] Xu J., Wang X., Sun K.S., Lin L., Zhou X. (2020). Parental self-medication with antibiotics for children promotes antibiotic over-prescribing in clinical settings in China. Antimicrob. Resist. Infect. Control.

[19] Lambert H., Chen M., Cabral C. (2019). Antimicrobial resistance, inflammatory responses: A comparative analysis of pathogenicities, knowledge hybrids and the semantics of antibiotic use. Palgrave Commun..

[20] Castrignanò E., Yang Z., Feil E.J., Bade R., Castiglioni S., Causanilles A., Gracia-Lor E., Hernandez F., Plósz B.G., Ramin P. (2020). Enantiomeric profiling of quinolones and quinolones resistance gene qnrS in European wastewaters. Water Res..

[21] Kasprzyk-Hordern B., Proctor K., Jagadeesan K., Edler F., Standerwick R., Barden R. (2021). Human population as a key driver of biochemical burden in an inter-city system: Implications for One Health concept. J. Hazard. Mater..

[22] Holton E., Kasprzyk-Hordern B. (2021). Multiresidue antibiotic-metabolite quantification method using ultra-performance liquid chromatography coupled with tandem mass spectrometry for environmental and public exposure estimation. Anal. Bioanal. Chem..

[23] Chopyk J., Chattopadhyay S., Kulkarni P., Claye E., Babik K.R., Reid M.C., Smyth E.M., Hittle L.E., Paulson J.N., Cruz-Cano R. (2017). Mentholation affects the cigarette microbiota by selecting for bacteria resistant to harsh environmental conditions and selecting against potential bacterial pathogens. Microbiome.

[24] Chopyk J., Allard S., Nasko D.J., Bui A., Mongodin E.F., Sapkota A.R. (2018). Agricultural Freshwater Pond Supports Diverse and Dynamic Bacterial and Viral Populations. Front. Microbiol..

[25] Wang H., Wang N., Wang B., Zhao Q., Fang H., Fu C., Tang C., Jiang F., Zhou Y., Chen Y. (2016). Antibiotics in Drinking Water in Shanghai and Their Contribution to Antibiotic Exposure of School Children. Environ. Sci. Technol..

