# Supplementary material for: Impacts of Antibiotic Residues in the Environment on Bacterial Resistance and Human Health in Eastern China: An Interdisciplinary Mixed-Methods Study Protocol
Source: Int J Environ Res Public Health. 2022 Jul 2;19(13):8145. doi: 10.3390/ijerph19138145 (PMC9266211; doi:10.3390/ijerph19138145)
Supplement: Supplementary file 1 [file ijerph-19-08145-s001.zip › ijerph-1751058-supplementary.pdf]

Supplementary Material

Table S1. Medicine Diary and family member antibiotic use records.

Table S1-a. Summary record of medication use for chronic diseases in Household.

Household no.______ **(20__Y___M___D~20__Y___M___D)**

S/N	Serial no.	Name	Gender	Age	Any chronic diseases	Type of chronic disease (please specify)	Any medication intakes for chronic diseases	Notes:
**1**					1.Yes 2.No		1.Yes 2.No	
2					1.Yes 2.No		1.Yes 2.No	
3					1.Yes 2.No		1.Yes 2.No	
4					1.Yes 2.No		1.Yes 2.No	
5					1.Yes 2.No		1.Yes 2.No	
6					1.Yes 2.No		1.Yes 2.No	
7					1.Yes 2.No		1.Yes 2.No	
8					1.Yes 2.No		1.Yes 2.No	
9					1.Yes 2.No		1.Yes 2.No	
10					1.Yes 2.No		1.Yes 2.No	

Table S1-b. Record of antibiotic use in household.

Name of respondent _______Household no.______ (20__Y___M___D~20__Y___M___D)

1.Reason	2.Date of intake		3.Drug name and serial no.	4. Delivery method	5. Dosage per take	6.Frequency	7.Source of drug	8. Regular intake or not	9. Side effects	Notes
	start	end								
①cold<br>②fever<br>③cough<br>④diarrhea<br>⑤urinary problems<br>⑥other__	__M<br>__D	__M<br>__D		①Tablet<br>②Capsule<br>③drug powder<br>④Injection<br>⑤IV<br>⑥Other	___tablet/pill/sachet/shot	① 1time/day<br>② 2times/day<br>③ 3times/day<br>④ 4times/day<br>⑤other_____	①Hospital prescription<br>②Clinic prescription<br>③Kept at home<br>④Self-bought from pharmacy<br>⑥Other_____	①Dosage on time as prescribed<br>②Less than prescribed everyday, less than____ dosage<br>③More than prescribed everyday, more than____ dosage<br>④Rarely or never follow prescription	①None<br>②Allergic reaction_____<br>③Digestive system reaction_____<br>④Other_____	
①cold<br>②fever<br>③cough<br>④diarrhea<br>⑤urinary problems<br>⑥other__	__M<br>__D	__M<br>__D		①Tablet<br>②Capsule<br>③drug powder<br>④Injection<br>⑤IV<br>⑥Other	___tablet/pill/sachet/shot	① 1time/day<br>② 2times/day<br>③ 3times/day<br>④ 4times/day<br>⑤other_____	①Hospital prescription<br>②Clinic prescription<br>③Kept at home<br>④Self-bought from pharmacy<br>⑥Other_____	①Dosage on time as prescribed<br>②Less than prescribed everyday, less than____ dosage<br>③More than prescribed everyday, more than____ dosage<br>④Rarely or never follow	①None<br>②Allergic reaction_____<br>③Digestive system reaction_____<br>④Other_____	
①cold<br>②fever<br>③cough<br>④diarrhea<br>⑤urinary problems<br>⑥other__	__M<br>__D	__M<br>__D		①Tablet<br>②Capsule<br>③drug powder<br>④Injection<br>⑤IV<br>⑥Other	___tablet/pill/sachet/shot	① 1time/day<br>② 2times/day<br>③ 3times/day<br>④ 4times/day<br>⑤other_____	①Hospital prescription<br>②Clinic prescription<br>③Kept at home<br>④Self-bought from pharmacy<br>⑥Other_____	①Dosage on time as prescribed<br>②Less than prescribed everyday, less than____ dosage<br>③More than prescribed everyday, more than____ dosage<br>④Rarely or never follow	①None<br>②Allergic reaction_____<br>③Digestive system reaction_____<br>④Other_____	

①cold<br>②fever<br>③cough<br>④diarrhea<br>⑤urinary problems<br>⑥other_	**__M<br>__D**	**__M<br>__D**		**①Tablet<br>②Capsule<br>③drug powder<br>④Injection<br>⑤IV<br>⑥Other**	**___tablet/pill/sachet/shot**	**① 1time/day<br>② 2times/day<br>③ 3times/day<br>④ 4times/day<br>⑤other_____**	**①Hospital prescription<br>②Clinic prescription<br>③Kept at home<br>④Self-bought from pharmacy<br>⑥Other_____**	**①Dosage on time as prescribed<br>②Less than prescribed everyday, less than____ dosage<br>③More than prescribed everyday, more than____ dosage<br>④Rarely or never follow**	**①None<br>②Allergic reaction_____<br>③Digestive system reaction_____<br>④Other_____**	

Appendix: Common antibiotic drug names and serial numbers.

**S/N**	**Name**		**S/N**	**Name**		**S/N**	**Name**
1.	Amoxicillin		13.	Cloxacillin		25.	Metronidazole
2.	Azithromycin dispersible tablets		14.	Phenoxymethyl penicillin		26.	Nitrofurantoin
3.	Cefixime dispersible tablets		15.	Piperacillin		27.	Sulfamethoxazole
4.	Ceftriaxone		16.	Procaine benzyl penicillin		28.	Cefuroxime axetil
5.	Cephalexin sustained release tablets		17.	Amikacin		29.	Levofloxacin Tablets<br>Levofloxacin Injection
6.	Cefaclor sustained release tablets		18.	Chloramphenicol		30.	Cefradine (Piperomycin VI, Cephalosporin VI)
7.	Ampicillin		19.	Ciprofloxacin		31.	Azithromycin soft capsules
8.	Benzathine penicillin		20.	Erythromycin		32.	Amoxicillin Clavulanate Potassium (Ammetidine)
9.	Penicillin G		21.	Clarithromycin		33.	Clavulanic acid
10.	Cephalexin		22.	Clindamycin		34.	Sulbactam

11.	Cefazolin		23.	Doxycycline		35.	
12.	Cefotaxime		24.	Gentamicin		36.	

Table S2. List of potential interviewees and proposed data collections.

Institutes	Personal	Requirement of Personal	Number pf Interviewees
Provincial Health Commission and food and drug administrative departments	related administrators	familiar with medical administration, administration on clinical medication, hospital infection-control, grassroot level	2-4
County level Health Commission	medical administration/primary managers	familiar with medical administration, administration on clinical medication, hospital infection-control, grassroot level	1-2
County level food and drug administrative departments	administrator	familiar with administration on clinical medication	1
County level environmental protection departments	administrator	familiar with water environmental treatment	1
County level agriculture, forestry and animal husbandry administrative departments	administrator	familiar with administration on usages of medication in breeding industry	1-2
County's People's hospitals	administrator	familiar with clinical practice, administration on medication	1-2
	chief of Pharmacy, clinical pharmacist	familiar with administration on medication, guidelines for the use of clinical antibiotics	1-2
	clinicians	Respiratory, Gastroenterology, Surgical, Pediatrics	4-5
	patients	Respiratory, Gastroenterology	2-4
Maternal and Child Hospital	administrator	familiar with clinical practice, administration on medication	1
	chief of Pharmacy, clinical pharmacist	familiar with administration on medication, guidelines for the use of clinical antibiotics	1
	clinicians	Respiratory, Gastroenterology, Surgical, Pediatrics	2
Private medical institutions	administrator	medical administration department or other (department?) that familiar with administration on medication	1

	department of Pharmacy	familiar with clinical practice, administration on medication	1
	medical staff	Doctors	2
Township agricultural technology stations	administrator	familiar with administration on usages of medication in breeding industry	1 person per township, 2 townships (1 from east side and 1 from west side)
Township health centres	administrator	familiar with clinical practice, administration on medication	1-2 persons per township, 2 townships
	chief of Pharmacy, clinical pharmacist	familiar with administration on medication, guidelines for the use of clinical antibiotics	1-2 persons per township, 2 townships
	GPs	Respiratory, Gastroenterology, Pediatrics	2-3 persons per township, 2 townships
	patients	Respiratory, Gastroenterology	2 persons per township, 2 townships
Private<br>Medical clinics	doctors	internal medicine	1 person per clinic, 2 clinics
Village clinics	village doctors		1 person per village clinic, 2 village clinics per township, 2 townships
	patient		1 group (4-5 persons) per village, 2 village clinics per township, 2 townships
Drug store	employee	Pharmacist or doctor	1 person per store, 2 stores
Farmers in breeding industry	residents	Breeding on fish, turtle, bullfrog, pig, chicken and duck	2 groups per township, 4-5 persons per groups
feed processing plant	worker	If there is any plant	1-2
